# Epidemiology and outcome predictors in 450 patients with hanging-induced cardiac arrest: a retrospective study

**DOI:** 10.3389/fneur.2023.1240383

**Published:** 2023-09-25

**Authors:** Marie Salvetti, Guillaume Schnell, Nicolas Pichon, Maleka Schenck, Pierrick Cronier, Sebastien Perbet, Jean-Baptiste Lascarrou, Christophe Guitton, Olivier Lesieur, Laurent Argaud, Gwenhael Colin, Bernard Cholley, Jean-Pierre Quenot, Hamid Merdji, Thomas Geeraerts, Michael Piagnerelli, Gwenaelle Jacq, Marine Paul, Jonathan Chelly, Louise de Charentenay, Nicolas Deye, Marc Danguy des Déserts, Guillaume Thiery, Marc Simon, Vincent Das, Frederic Jacobs, Charles Cerf, Julien Mayaux, Pascal Beuret, Abdelkader Ouchenir, Antoine Lafarge, Bertrand Sauneuf, Cedric Daubin, Alain Cariou, Stein Silva, Stephane Legriel

**Affiliations:** ^1^Medical-Surgical Intensive Care Unit, Centre Hospitalier de Versailles—Site André Mignot, Le Chesnay, France; ^2^Medical-Surgical Intensive Care Unit, GH Le Havre, Le Havre, France; ^3^Medical-Surgical Intensive Care Unit, CHU de Limoges, Limoges, France; ^4^AfterROSC, Paris, France; ^5^Médecine Intensive Réanimation, Hôpital de Hautepierre, Hôpitaux Universitaires de Strasbourg, Strasbourg, France; ^6^Intensive Care Unit, Sud-Francilien Hospital Center, Corbeil-Essonnes, France; ^7^Department of Perioperative Medicine, University Hospital of Clermont-Ferrand, 58 Rue Montalembert, Université Clermont Auvergne, CNRS, INSERM, GReD, Clermont-Ferrand, France; ^8^Medicine Intensive Reanimation, University Hospital, Nantes, France; ^9^Medical-Surgical Intensive Care Unit, Centre Hospitalier du Mans, Le Mans, France; ^10^Intensive Care Unit, Saint-Louis Hospital, La Rochelle, France; ^11^Medical Intensive Care Unit, Hospices Civils de Lyon, Edouard Herriot Teaching Hospital, Lyon, France; ^12^Medical-Surgical Intensive Care Unit, La Roche-sur-Yon District Hospital Centre, La Roche-sur-Yon, France; ^13^Hôpital Européen Georges Pompidou, Assistance Publique-Hôpitaux de Paris, Université Paris Cité et Service d'Anesthésie-Réanimation Médecine Péri Opératoire, Paris, France; ^14^Service de Médecine Intensive-Réanimation, CHU Dijon Bourgogne, Dijon, France; ^15^Faculté de Médecine, Hôpitaux Universitaires de Strasbourg, Nouvel Hôpital Civil, Service de Médecine Intensive Réanimation, Université de Strasbourg (UNISTRA), Strasbourg, France; ^16^UMR 1260, Regenerative Nano Medicine, INSERM, Fédération de Médecine Translationnelle de Strasbourg (FMTS), Université de Strasbourg, Strasbourg, France; ^17^Department of Anaesthesiology, Critical Care and Perioperative Medicine, Toulouse University Hospital, Toulouse, France; ^18^Intensive Care Unit, Marie-Curie Teaching Hospital, Université Libre de Bruxelles, Charleroi, Belgium; ^19^Intensive Care Unit, Groupe Hospitalier Sud Ile de France, Melun, France; ^20^Medical Intensive Care Unit, Lariboisière University Hospital, APHP, Paris, France; ^21^INSERM UMR-S 942, Lariboisière Hospital, Paris, France; ^22^Intensive Care Unit, Clermont Tonnerre Military Hospital, Brest, France; ^23^Medical-Surgical Intensive Care Unit, Saint-Étienne University Hospital, Saint-Étienne, France; ^24^Department of Intensive Care, Cliniques du Sud-Luxembourg of Arlon, Arlon, Belgium; ^25^Medical-Surgical Intensive Care Unit, Centre Hospitalier Intercommunal André Grégoire, Montreuil, France; ^26^Medical Intensive Care Unit, Beclère Teaching Hospital, Clamart, France; ^27^Department of Intensive Care, Foch Hospital, Suresnes, France; ^28^Department of Pulmonology and Intensive Care, Pitié-Salpêtrière Teaching Hospital, Paris, France; ^29^Department of Intensive and Continuous Care, Roanne Hospital, Roanne, France; ^30^Medical Intensive Care Unit, Louis Pasteur Hospital, Chartres, France; ^31^Medical Intensive Care Unit, Saint Louis Teaching Hospital, Assistance Publique-Hôpitaux de Paris, Paris, France; ^32^General Intensive Care Unit, Cotentin Public Hospital Centre, Cherbourg-en-Cotentin, France; ^33^Medical Intensive Care Unit, Caen Teaching Hospital, Caen, France; ^34^Medical Intensive Care Unit, Cochin University Hospital, Assistance Publique-Hôpitaux de Paris and Université de Paris, Paris, France; ^35^INSERM U970, Paris Cardiovascular Research Centre, Paris, France; ^36^Critical Care Unit, University Teaching Hospital of Purpan, Toulouse, France; ^37^UVSQ, INSERM, CESP, PsyDev Team, Paris-Saclay University, Villejuif, France

**Keywords:** near-hanging, intensive care unit, coma/therapy, outcome, cardiopulmonary resuscitation

## Abstract

**Background:**

Cardiac arrest is the most life-threatening complication of attempted suicide by hanging. However, data are scarce on its characteristics and outcome predictors.

**Methods:**

This retrospective observational multicentre study in 31 hospitals included consecutive adults admitted after cardiac arrest induced by suicidal hanging. Factors associated with in-hospital mortality were identified by multivariate logistic regression with multiple imputations for missing data and adjusted to the temporal trends over the study period.

**Results:**

Of 450 patients (350 men, median age, 43 [34–52] years), 305 (68%) had a psychiatric history, and 31 (6.9%) attempted hanging while hospitalized. The median time from unhanging to cardiopulmonary resuscitation was 0 [0–5] min, and the median time to return of spontaneous circulation (ROSC) was 20 [10–30] min. Seventy-nine (18%) patients survived to hospital discharge. Three variables were independently associated with higher in-hospital mortality: time from collapse or unhanging to ROSC>20 min (odds ratio [OR], 4.71; 95% confidence intervals [95%CIs], 2.02–10.96; *p* = 0.0004); glycaemia >1.4 g/L at admission (OR, 6.38; 95%CI, 2.60–15.66; *p* < 0.0001); and lactate >3.5 mmol/L at admission (OR, 6.08; 95%CI, 1.71–21.06; *p* = 0.005). A Glasgow Coma Scale (GCS) score of >5 at admission was associated with lower in-hospital mortality (OR, 0.009; 95%CI, 0.02–0.37; *p* = 0.0009).

**Conclusion:**

In patients with hanging-induced cardiac arrest, time from collapse or unhanging to return of spontaneous circulation, glycaemia, arterial lactate, and coma depth at admission were independently associated with survival to hospital discharge. Knowledge of these risk factors may help guide treatment decisions in these patients at high risk of hospital mortality.

## Introduction

Suicide is a public health issue that is responsible for ~700,000 deaths each year worldwide and 1.3% of all deaths in 2019 (1). Hanging is a common and particularly lethal suicide method used chiefly by young men with psychiatric or addictive comorbidities (2–5). Hanging can result in various types of injury and can lead to cardiac arrest (CA) via several mechanisms. In recent studies, cardiac arrest was present in initial management in 20%−60% of patients (4–7). The interruption of cervical blood flow by hanging results in early brain ischaemia, which is worsened by CA (2, 4, 5). Various cervical (2, 3, 5, 8–11) and thoracic (2, 3, 9) structures may be injured. Patients may also suffer damage to the heart, which is usually reversible, and/or acute pulmonary oedema (8, 12, 13).

Mortality and residual functional impairments are common after near-hanging. Death may occur immediately if resuscitation is not provided or after hospital management. In-hospital mortality rates have varied widely, from ~10% to nearly 80%, mainly due to the selection of patient characteristics (4, 5, 14, 15). One of the main determinants of mortality and residual neurological impairment is the occurrence of hanging-induced CA (4, 7, 9, 11, 14, 16). In a retrospective study of 886 ICU patients with hanging injuries, 450 patients had cardiac arrest, which was strongly associated with mortality (OR 19.50; 95%CI, 7.21–60.90) (17). This high rate of mortality is probably ascribable to CA-related systemic ischaemia-reperfusion syndrome with initial cardiogenic, vasoplegic hemodynamic failure, and anoxic brain injury (18).

To date, few studies have focussed specifically on outcome predictors in patients with hanging-induced CA. Therefore, this multicentre retrospective study aimed to describe patient characteristics and hospital survival after hanging-induced CA and identify predictors of in-hospital mortality.

## Materials and methods

This study complies with the standards set by the French and Belgian legislation on retrospective clinical research aimed at protecting the confidentiality of personal data. The study protocol was approved by the ethics committee (*Comité de Protection des Personnes de Paris, Ile de France XI*, 13 September 2012, #XI/12061) and is registered on ClinicalTrials.gov (#NCT04096976). Survivors retrospectively received a written consent form as soon as they recovered decision-making competency; if they refused consent, they were excluded from the registry. All procedures involving the patients complied with the ethical standards of our institutional and national research committees and with the 1964 Declaration of Helsinki and its later amendments.

### Study population

Consecutive adults admitted to one of the 31 university or university-affiliated ICUs in France and Belgium after successfully resuscitating people after a suicidal near-hanging injury between February 1992 and May 2014 were identified. Among them, patients older than 18 years who experienced CA, followed by the return of spontaneous circulation (ROSC), were included in the present study.

### Patient and public involvement

It was not appropriate or possible to involve patients and/or the public in the design, conduct, report, or disseminate plans of our research.

### Data collection

As previously described (17), we used a standardized form for each patient to collect demographic data, medical history, and the characteristics of the hanging-induced CA according to the Utstein-style guidelines (19).

### Study outcomes

Hospital mortality was the primary outcome measure. We also described patient characteristics and identified independent predictors of in-hospital mortality as secondary outcomes.

### Statistical analysis

Quantitative parameters were described as a median in the form of interquartile range (IQR) and qualitative parameters in the form of numbers (percentage). We compared the categorical variables using Fisher's exact test and compared the continuous variables using the Wilcoxon rank-sum test. We ordered categorical variables using the chi-square and the Kruskal-Wallis tests. We then performed logistic regression to identify associations between factors and hospital mortality. Continuous variables were checked for log-linearity. Non-log-linear variables were transformed into dummy variables according to their inflection point or median value. Non-collinear variables that yielded *p*-values <0.05 by univariate analysis or were clinically relevant were considered for inclusion into a multivariable model.

Stepwise model selection guided by the Akaike Information Criterion was performed. Variables tested were age; male sex; time from collapse or unhanging to ROSC; asystole as first recorded rhythm; GCS score, body temperature, pulse oximetry, glycaemia, and lactate at admission; and the total number of organ failures on day 1. Time of management (1992–1995, 1996–2000, 2001–2005, 2006–2010, or 2011–2014) and Simplified Acute Physiology Score (SAPS) II score on day one after admission were used for adjustment. Missing data were handled under the data-missing-at-random hypothesis using multiple imputations by chained equations (52 imputations, 10 iterations). Associations of factors with hospital mortality were reported as ORs with their 95%CIs. All tests were two-sided, and *p*-values of <0.05 were considered significant.

The analyses were performed using the R statistical programme, version 4.1.0 (R Foundation for Statistical Computing, Vienna, Austria).^1^

## Results

**Figure 1:**
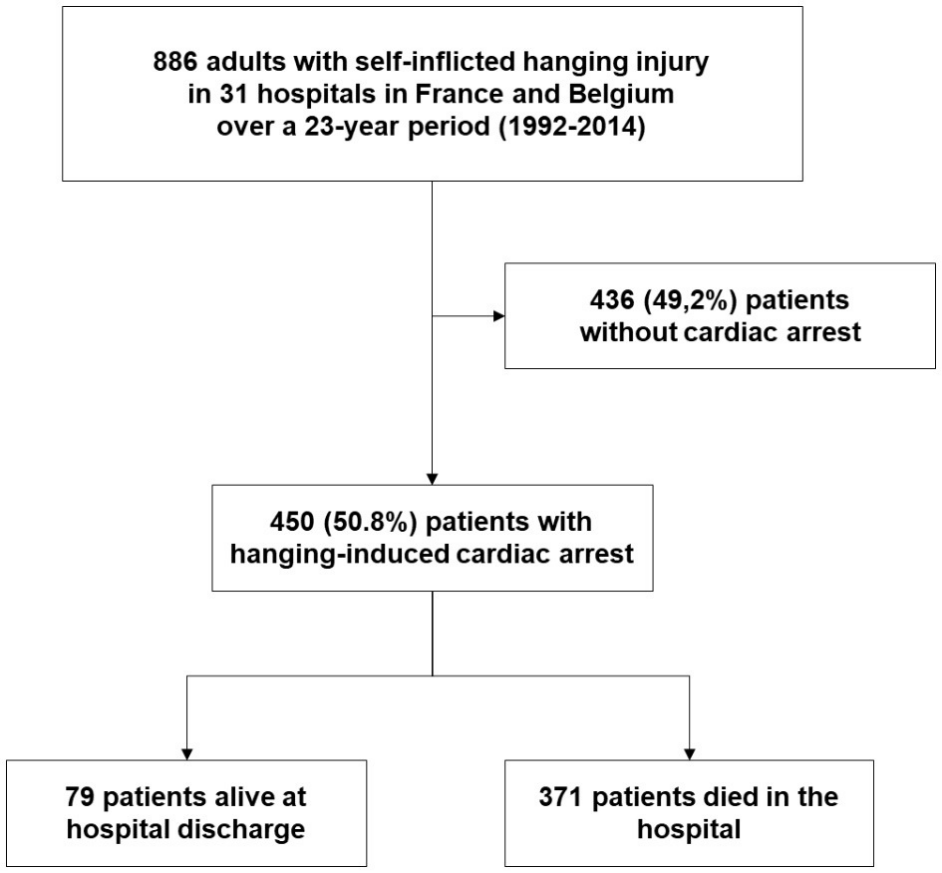
Patient flow chart.

Of the 886 patients admitted to the 31 participating hospitals after near-hanging during the 23-year study period, 450 (51%) experienced CA and were included in this study (Figure 1).

### Patient characteristics and on-scene management of cardiac arrest

Table 1 reports the main patient characteristics. Overall, presumed fatal comorbidities were very rare, but approximately two-thirds of patients had psychiatric diagnoses. For most patients, hanging was the first attempt at suicide. Cardiac arrest usually occurred at home, and approximately half of the patients received basic resuscitation using an automated external defibrillator before the arrival of emergency services. Asystole was the most common first recorded rhythm. Overall, a quarter of the patients received at least one external electric shock, and approximately three-quarters received adrenaline with a median cumulative dose of 3 mg before ROSC. The median time from collapse or unhanging to ROSC was 20 [10–30] min.

**Table 1:** Cardiac arrest the Utstein-style guidelines criteria in 450 patients with hanging-induced cardiac arrest.

**Utstein criteria**	**n (%) and median [IQR]**
Age, years, median [IQR] (md = 3)	43 [34–52]
Male sex, *n* (%)	350 (77.8)
Location at cardiac arrest, *n* (%)	
*Home*	349 (77.6)
*Public place*	27 (6)
*Hospital*	31 (6.9)
*Correctional facility*	31 (6.9)
*Other*	12 (2.7)
First recorded rhythm, *n* (%) (md = 1)	
*Asystole*	248 (55.1)
*Pulseless electrical activity*	21 (4.7)
*Ventricular fibrillation/ventricular tachycardia*	75 (16.7)
*Other*	105 (23.3)
Number of defibrillations before ROSC, median [IQR] (md = 1)	0 [0–0]
Total adrenaline dose before ROSC, mg, median [IQR] (md = 3)	3 [0–5]
Time from collapse/unhanging, min, median [IQR]	
*to CPR (no-flow) (md = 4)*	0 [0–5]
*to ROSC (no-flow + low-flow) (md = 80)*	20 [10–30]
Lactate at admission, mmol/L, median [IQR] (md = 143)	7 [4–10.4]
Targeted temperature management (32–36°C), *n* (%)	217 (48.2)

IQR, interquartile range; md: missing data; CPR, cardiopulmonary resuscitation; ROSC, return of spontaneous circulation.

### In-hospital management of hanging-induced cardiac arrest

At admission, 71% of patients underwent cerebral and cervical computed tomography (CT), 5% cervical-spine radiography, and 3% cerebral and cervical magnetic resonance imaging (MRI). A bony cervical lesion with spinal injury was found in 5% of the 343 patients with cervical imaging and a vascular cervical lesion in 2% of those with vascular imaging. Targeted temperature management was used in 217 (48%) patients for a median effective duration of 24 [23–24] h.

### Outcomes

Table 2 reports factors associated with in-hospital mortality. Mechanical ventilation was used for 4 [2–7] days, and the median length of hospital stay was 5 [2–9] days. Overall, in-hospital mortality was 82% (371/450).

**Table 2:** Factors associated with in-hospital mortality in 450 patients after hanging-induced cardiac arrest.

**Factors associated with in-hospital mortality**	**N (%) or Median [interquartile range]**		**Univariate analysis**		
	**Patients who died in the hospital**, ***N*** = **371**	**Patients alive at hospital discharge**, ***N*** = **79**	**OR**	**95%CI**	***p*** **-value**
**Demographic and patients characteristics**					
Age, years, median [IQR]	44 [35–54]	41 [32–46]	1.03	1.01–1.04	0.007
Male sex, *n* (%)	281 (75.7)	69 (87.3)	0.45	0.22–0.92	0.03
Psychiatric comorbidity, *n* (%)	251 (67.7)	54 (68.4)	0.97	0.57–1.63	0.9
Alcohol abuse, *n* (%)	82 (22.1)	21 (26.6)	0.78	0.45–1.37	0.39
Other substance abuse, *n* (%)	25 (6.7)	11 (13.9)	0.45	0.21–0.95	0.04
Number of previous suicide attempts, median [IQR]	0 [0–1]	0 [0–1]	1	0.83–1.20	0.98
Previous hanging attempt, *n* (%)	21 (5.7)	7 (8.9)	0.62	0.25–1.51	0.29
Absence of presumed fatal comorbidity^*^, *n* (%)	364 (98.1)	77 (97.5)	1.35	0.28–6.63	0.71
**Cardiac arrest characteristics**					
Out-of-hospital hanging-induced cardiac arrest, *n* (%)	346 (93.3)	72 (92.4)	1.14	0.45–2.87	0.79
Use of AED, *n* (%)	194 (52.4)	22 (27.9)	2.86	1.68–4.86	0.0001
CPR before emergency service arrival, *n* (%)	169 (45.7)	41 (52.6)	0.76	0.47–1.24	0.27
Asystole as first recorded rhythm, *n* (%)	217 (58.5)	31 (39.2)	2.18	1.33–3.58	0.002
Number of defibrillations before ROSC, median [IQR]	0 [0–1]	0 [0–0]	2.9	1.48–5.68	0.002
Adrenaline dose before ROSC, mg, median [IQR]	3 [1–6]	0 [0–0]	2.38	1.85–3.06	< 0.0001
Time from collapse or unhanging, median [IQR]					
to CPR (no-flow), min, median [IQR]	0 [0–5]	0 [0–0]	1.14	1.05–1.25	0.002
to ROSC (no-flow + low-flow), min, median [IQR]	23 [13–30]	3 [1–15]	1.11	1.08–1.15	< 0.0001
**Clinical, laboratory, and imaging findings at admission**					
Glasgow Coma Scale score, median [IQR]	3 [3–3]	3 [3–5]	0.4	0.28–0.56	< 0.0001
Body temperature, °C, median [IQR]	35.5 [34.5–36.7]	36.6 [35.8–37.2]	0.78	0.68–0.90	0.0006
Systolic blood pressure (mmHg), median [IQR]	115 [94–140]	118 [100–134]	1	0.99–1.01	0.97
Pulse oximetry, %, median [IQR]	99 [96–100]	99 [98–100]	0.81	0.71–0.93	0.002
Lactate (mmol/L), median [IQR]	7.9 [5–11]	2.7 [1.4–3.6]	1.43	1.26–1.62	< 0.0001
Glycemia, g/L, median [IQR]	2.3 [1.5–2.9]	1.2 [1–1.4]	5.4	3.27–8.94	< 0.0001
PaO_2_/FiO_2_, mmHg, median [IQR]	322 [203–457]	300 [191–457]	1	1.00–1.00	0.25
PaCO_2_, mmHg, median [IQR]	38 [31–45]	37 [34–44]	0.99	0.97–1.01	0.5
Serum sodium, mmol/L, median [IQR]	139 [137–142]	140 [138–142]	0.98	0.93–1.04	0.56
Cervical spine and/or vascular injury on CT or MRI, *n* (%)	22 (8.1)	1 (1.4)	6.27	0.83–47.3	0.08
**Severity scores on day one after hospital admission**					
SAPS II score, median [IQR]	62 [52–73]	49 [39–62]	1.07	1.05–1.09	< 0.0001
Total LODS score, median [IQR]	8 [7–10]	6 [5–7]	1.93	1.60–2.33	< 0.0001
Total number of organ failures,^**^ median [IQR]	3 [3–4]	2 [2–3]	2.99	2.16–4.14	< 0.0001
Neurological failure^**^, *n* (%)	369 (99.5)	74 (93.7)	12.5	2.37–65.5	0.003
Cardiovascular failure^**^, *n* (%)	178 (48)	15 (19)	3.94	2.16–7.16	< 0.0001
Renal failure^**^, *n* (%)	238 (64.2)	18 (22.8)	6.06	3.44–10.7	< 0.0001
Respiratory failure^**^, *n* (%)	357 (96.2)	73 (92.4)	2.1	0.78–5.63	0.14
Hematological failure^**^, *n* (%)	18 (4.9)	4 (5.1)	0.96	0.31–2.91	0.94
Hepatic failure^**^, *n* (%)	36 (9.7)	1 (1.3)	8.38	1.13–62.1	0.04
**Targeted temperature management** (32–36°C), *n* (%)	182 (49)	35 (44.3)	1.21	0.74–1.97	0.44

IQR, interquartile range; OR, odds ratio; 95%CI, 95% confidence interval; AED, automatic external defibrillator; CPR, cardiopulmonary resuscitation; ROSC, return of spontaneous circulation; CT, computed tomography; MRI, magnetic resonance imaging; SAPS, Simplified Acute Physiology Score; LODS, Logistic Organ Dysfunction System.

^*^according to the McCabe classification.

^**^according to the Logistic Organ Dysfunction System score.

Brain death occurred in 120 (27%) patients at a median time of 4 [2–5] days after CA. A decision to withdraw life-sustaining interventions was made for 162 (36%) patients at a median time of 5 [3–8] days after hanging. Of the 79 patients who survived to hospital discharge, 69 (87%) had a favorable neurological outcome defined as a Cerebral Performance Category (CPC) score of 1 or 2.

### Predictors of hospital mortality

**Figure 2:**
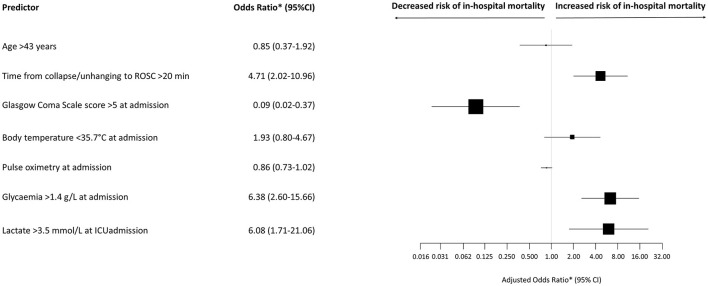
Odds ratios for hospital mortality. ^*^Adjusted according to time of management (1992–1995, 1996–2000, 2001–2005, 2006–2010, or 2011–2014) and SAPS II score on day 1 after ICU admission. Data marker sizes reflect the relative size of each covariate. Error bars indicate 95% confidence intervals of odds ratios. ROSC, return of spontaneous circulation; ICU, intensive care unit.

Using multivariate analysis after missing data imputation (Figure 2), we found that three variables were independently associated with higher hospital mortality: time from collapse or unhanging to ROSC >20 min (OR, 4.71; 95%CI, 2.02–10.96; *p* = 0.0004); glycaemia >1.4 g/L at admission (OR, 6.38; 95%CI, 2.60–15.66; *p* < 0.0001); and lactate >3.5 mmol/L at admission (OR, 6.08; 95%CI, 1.71–21.06; *p* = 0.005). In addition, a GCS score of >5 at admission was associated with lower in-hospital mortality (OR, 0.009; 95%CI, 0.02–0.37; *p* = 0.0009).

## Discussion

Of the 450 patients with hanging-induced CA, 79 (18%) were discharged alive from the hospital. Four factors were independently associated with the risk of hospital death: time from collapse or unhanging to ROSC >20 min, glycaemia >1.4 g/L at admission, and arterial lactate >3.5 mmol/L at admission were associated with higher hospital mortality, and a GCS score of >5 at admission was associated with lower hospital mortality.

Our 450 patients with CA constituted approximately half of the patients admitted to 31 hospitals after attempted suicide by hanging, which is within the previously reported 20%−60% range (4–7). The main characteristics of our patients were consistent with earlier reports (2–6, 20–23).

Our cohort had a higher frequency of basic CPR delivered before emergency-service arrival, i.e., 47% compared to 10%−30% (5, 20–23) in keeping with the shorter no-flow time and lower lactate level in our patients (5, 12, 21, 22). Further, there was an unexpectedly higher proportion of patients with an initial shockable rhythm compared to earlier studies (5, 20–23). Few studies have reported the frequency of cardiovascular or hemodynamic failure after hanging-induced cardiac arrest. In our population, two-fifths of patients had cardiovascular failure according to the LODS score. Previously reported serum lactate levels at admission were often higher than that reported in our study, ranging from 5 to 13 mmol/L. Kidney failure, often with shock, occurred in 30%−50% of patients with CA due to hanging or other causes, compared to 58% in our study (24, 25). Cervical CT or MRI were infrequently performed and rarely showed spinal or vascular injuries in our patients, in accordance with other data (2, 5, 9, 10). The devastating consequences of missing such injuries are prompting the increased use of contrast-enhanced cervical CT after hanging (3, 7, 9, 11).

Only 18% of our patients were discharged alive from the hospital. However, among them, 87% had a favorable neurological outcome with a CPC score of 1 or 2 at hospital discharge. Two studies from South Korea found noticeably higher survival rates of 43% and 52%, respectively, but with a persistent vegetative state in over 80% of survivors (21, 22). In an Australian study, 3% of patients survived to discharge, and most had limited or no disability (20). These results suggest differences across countries in criteria for treatment-limitation decisions. However, a recent study from North America had a 29% hospital survival rate with satisfactory neurological outcomes in most survivors (5).

A time from collapse or unhanging to ROSC longer than 20 min predicted hospital mortality in our study and another study (11). No-flow and low-flow times are well-established predictors of mortality after CA (26, 27). The two other predictors of higher mortality were high serum lactate and high glycaemia in keeping with other studies. Tight blood glucose control has been demonstrated to improve the neurological prognosis after CA (4). Further, a less severe consciousness impairment at admission was associated with hospital survival; the cut-off was a GCS score of >5, compared to 3–6 in other studies (5, 12, 22).

The retrospective design of our study is a major limitation that led to a substantial proportion of missing data for some variables. However, we used the multiple-chain equation method to counterbalance this weakness. We hypothesize that the longer the hanging time, the lower the probability of reaching ROSC. However, the exact duration of hanging-induced CA is generally unknown, and the no-flow and low-flow times should therefore be taken as minimum values. This limitation is inherent in our type of population. However, our study only included patients who achieved ROSC and could consequently be secondarily managed in the intensive care unit. Thus, while the delay between hanging and unhanging is unknown, we believe that the effect of this delay on ROSC is unlikely in our population. Moreover, whereas 71% of patients underwent cerebral and cervical computed tomography, we cannot provide information on the repetition of these explorations during real-time patient management to assess for structural damage and/or diffuse axonal injury. Practices may have changed over the 23-year study period. Moreover, the study period ended in 2014, and since then, further changes have occurred, notably regarding the use of targeted temperature management. However, in our study, targeted temperature management was not significantly associated with hospital survival. Finally, we did not obtain follow-up data after hospital discharge.

To conclude, in this retrospective multicentre observational study of 450 patients admitted to the hospital after hanging-induced CA, time from collapse or unhanging to ROSC, glycaemia, arterial lactate rate, and the GCS at admission were independently associated with hospital survival. Knowledge of these risk factors may help guide treatment decisions for this population of patients at high risk of hospital mortality.

## Antigone investigators

Nicolas Girard (La Rochelle), Martin Cour (Lyon), Adriaan Prisacariu (Arlon), Auguste Dargent (Dijon), Ferhat Meziani (Strasbourg), Thibaut Baudic (Brest), Philippe Vignon (Limoges), Candice Belony (Le Chesnay), Charlene Leparq (Le Chesnay), Cecile Carre (Le Chesnay), Pauline Moriss (Le Chesnay), Emmanuelle Noel (Le Chesnay), Quentin De Roux (Creteil), Matthieu Resche-Rigon (Paris), Thomas Rossignol (Le Mans), Bruno Megarbane (Paris), Alexis Soummer (Suresnes), Alexandre Demoule (Paris), Pierre Kalfon (Chartres), Charlotte Martin (Toulouse), Elie Azoulay (Paris).

## Data availability statement

Publicly available datasets were analyzed in this study. Ethical restrictions apply to the availability of these data regarding participant privacy prohibiting us from making the entire data set publicly available. However, after publication, data will be available to any researcher who provides a methodologically sound study proposal that is approved by the central study team. Proposals can be submitted to the Versailles Hospital (slegriel@ght78sud.fr). Individual patients and hospitals will not be identifiable in any released data and all appropriate information governance protocols will be followed.

## Ethics statement

The studies involving humans were approved by Comité de Protection des Personnes de Paris, Ile de France XI. The studies were conducted in accordance with the local legislation and institutional requirements. Written informed consent for participation was not required from the participants or the participants' legal guardians/next of kin in accordance with the national legislation and institutional requirements.

## Author contributions

SL: conceived, designed, supervised the trial, coordinated the data collection, and performed the statistical analysis. SL, LC, MD, GS, NP, J-BL, JC, MSi, and GJ: collected the data. SL and MSa: analyzed and interpreted the data and wrote the first draft of the manuscript. All authors revised the manuscript for important intellectual content and approved the final version of the manuscript.

## Footnotes

^1^http://www.R-project.org (accessed 20 December, 2022).
